# Atypical manifestations of leishmaniasis in the state of Mato Grosso: a clinical-epidemiological study at the Júlio Müller University Hospital (2013-2023)

**DOI:** 10.1590/0037-8682-0440-2025

**Published:** 2026-03-27

**Authors:** Bianca Coelho Damin Ribeiro, Tatiana Fortes de Oliveira, Tiago Rodrigues Viana, Giovana Volpato Pazin Feuser, Fernanda Ferreira Fernandes, Marcia Hueb

**Affiliations:** 1 Universidade Federal de Mato Grosso, Hospital Universitário Júlio Müller, Cuiabá, MT, Brasil.; 2 Hospital Universitário Júlio Müller, Ambulatório 3, Cuiabá, MT, Brasil.; 3 Universidade Federal de Mato Grosso, Departamento Clínica Médica, Cuiabá, MT, Brasil.; 4 Universidade Federal de Mato Grosso, Faculdade de Medicina, Cuiabá, MT, Brasil.

**Keywords:** Leishmaniasis, Leishmania, Atypical leishmaniasis, Mato Grosso, Brazil

## Abstract

**Background:**

Leishmaniasis is a neglected tropical infectious disease caused by protozoa of the *Leishmania* genus and transmitted by the bite of female sand flies. It may present with various clinical manifestations depending on species, parasite load, and host immunity. Recently, a geographic expansion of the disease has been observed, along with variations in clinical manifestations.

**Methods:**

We aimed to better define this clinical entity in the Brazilian context by describing leishmaniasis cases with atypical clinical manifestations treated at the Júlio Müller University Hospital in Cuiabá, Mato Grosso, between 2013 and 2023.

**Results:**

During the study period, 1,041 cases of cutaneous and mucosal leishmaniasis and 51 suspected cases of visceral leishmaniasis were recorded, of which 47 were classified as atypical (4.5%). Atypical presentations included localized cutaneous forms with unusual morphology (91.5%), mucocutaneous forms (4.2%), one case resembling post-kala-azar dermal leishmaniasis (2.1%), and one case of mucocutaneous lesions occurring after visceral leishmaniasis (2.1%), both of which are considered atypical in Brazil, where there is no endemic description of post-kala-azar dermal leishm aniasis. Most cases involved men aged 50-59 years, who were predominantly rural workers. These cases primarily originated from the Cerrado biome. The lesions had a median evolution of 120 d and mainly affected the lower limbs and face. Uncommon symptoms included fever, weight loss, and hepatosplenomegaly. Molecular identification of the etiological agent contributed to the diagnosis of selected cases.

**Conclusions:**

This study highlights the importance of recognizing atypical clinical manifestations of leishmaniasis to improve disease diagnosis and management.

## INTRODUCTION

Leishmaniasis is a group of tropical parasitic infectious diseases recognized as neglected diseases^1^. It is caused by a protozoan of the genus *Leishmania* and is transmitted through the bites of female sand flies^2^. Clinical manifestations range from Cutaneous Leishmaniasis (CL), which includes localized, disseminated, and mucosal forms, to Visceral Leishmaniasis (VL), which can be fatal. The severity depends on the parasite species, parasite load, and host immune response^1^ ^,^ ^3^ ^,^ ^4^.

Geographically, different forms of leishmaniasis are present in 102 countries, of which 87 are endemic, with an incidence of approximately 1.3 million new cases annually. Cutaneous Leishmaniasis, with approximately 1 million annual cases, is mainly concentrated in countries, such as Afghanistan, Brazil, and Iran. By contrast, VL causes approximately 300,000 new cases per year, most of which occur in countries, such as Bangladesh and Brazil^4^ ^,^ ^5^.

Twelve species of *Leishmania* that affect the skin have been identified in the United States, seven of which are found in Brazil. CL is prominent in Brazil owing to its widespread distribution^6^. Clinically, CL usually manifests as a painless ulcer with raised edges and a granular base in exposed areas of the skin. It is rounded or oval, measuring a few millimeters to a few centimeters, with an erythematous and infiltrated base^7^. Cases of CL considered atypical, that is, cases of leishmaniasis that do not fit the classical forms described, have been reported. Such atypical manifestations may occur in up to 20% of cases, producing acneiform, eczematous, sporotrichoid, erysipeloid, or zosteriform lesions^2^ ^,^ ^4^.

Leishmaniasis is expanding beyond its classic manifestations and geographic areas, with emerging clinical variations and genotypes^2^ ^,^ ^8^. In Africa and the Indian subcontinent, *Leishmania donovani*, a species responsible for causing VL, can cause skin manifestations after treatment, known as post-kala-azar dermal leishmaniasis (PKDL)^9^. Although rare and mostly associated with a diagnosis of human immunodeficiency virus infection, CL cases caused by *Leishmania infantum chagasi* have been reported in Brazil, particularly in the Northeast^9^.

Given these variations, relying solely on clinical criteria to reach a diagnosis can be flawed. The diversity of human genetics and variety of *Leishmania* species lead to different forms of the disease, making diagnosis and treatment challenging, especially in atypical forms^8^. Therefore, more detailed studies are necessary to better characterize this nosological entity and optimize its clinical management.

Despite the evidence of an increase in atypical cases in Mato Grosso and Brazil, there are few reports on this subject in the scientific literature. This study aimed to contribute to the detailed characterization necessary to define atypical leishmaniasis, thereby establishing more specific clinical and laboratory standards to distinguish it from other forms.

## METHODS

This retrospective descriptive study analyzed patients with leishmaniasis presenting with atypical clinical manifestations treated between 2013 and March 2023 at the Referral Service for Diagnosis and Treatment of Leishmaniasis at the Júlio Müller University Hospital (HUJM) in Cuiabá, Mato Grosso. This study was conducted according to international ethical guidelines for biomedical research involving human beings (Resolution 466/12 CNS, 2012) and was approved by the Research Ethics Committee, CEP/HUJM, approval number 5,726,648 (CAE 64330822.0.0000.5541).

Sociodemographic and clinical data extracted from medical records, such as municipality of origin, sex, age, symptoms, lesion characteristics, and test results, were entered into an Excel spreadsheet.

A map of Mato Grosso was used to represent the number of detected cases in relation to the reported municipality of infection. Using the Brazilian Institute of Geography and Statistics cartographic base to delimit municipalities and biomes, the data were processed, treated, and exported to QGIS software, finalized in SHAPEFILE format, referenced to the SIRGAS 2000 Datum, and compiled into a digital cartographic base at a scale of 1:4,000,000^10^.

Patients with *Leishmania* identified by any of the parasitological methods (culture, scraping smears, tissue impression on slides, and visualization by histopathology) or molecular methods (polymerase chain reaction [PCR]) were included in the study. Additionally, species typification was performed whenever possible.

Patients with classic clinical manifestations or those without laboratory confirmation of leishmaniasis were excluded from the analysis. Patients with a confirmed diagnosis of human immunodeficiency virus infection were excluded, as were those with other conditions associated with significant immunosuppression. Patients with characteristics of disseminated leishmaniasis were also excluded.

Cases were considered atypical when manifestations differed from the usual ones, such as ulcers without defined borders or infiltration, nonulcerated lesions, the presence of *Leishmania infantum* in skin lesions, systemic manifestations in CL, or skin lesions associated with VL.

## RESULTS

A total of 1,041 cases of CL were reported at HUJM between 2013 and March 2023, of which 47 (4.5%) were considered atypical.

The typical form of leishmaniasis was present in approximately 51-140 cases per year, with peaks in 2015 and 2019 (140 cases each) and a progressive reduction from 2020 onward, reaching 17 cases in the partial data for 2023. The atypical form showed an annual variation between zero and 13 cases, with the highest number recorded in 2018 (12 cases) and values below 10 cases per year in the other periods.

**FIGURE 1:**
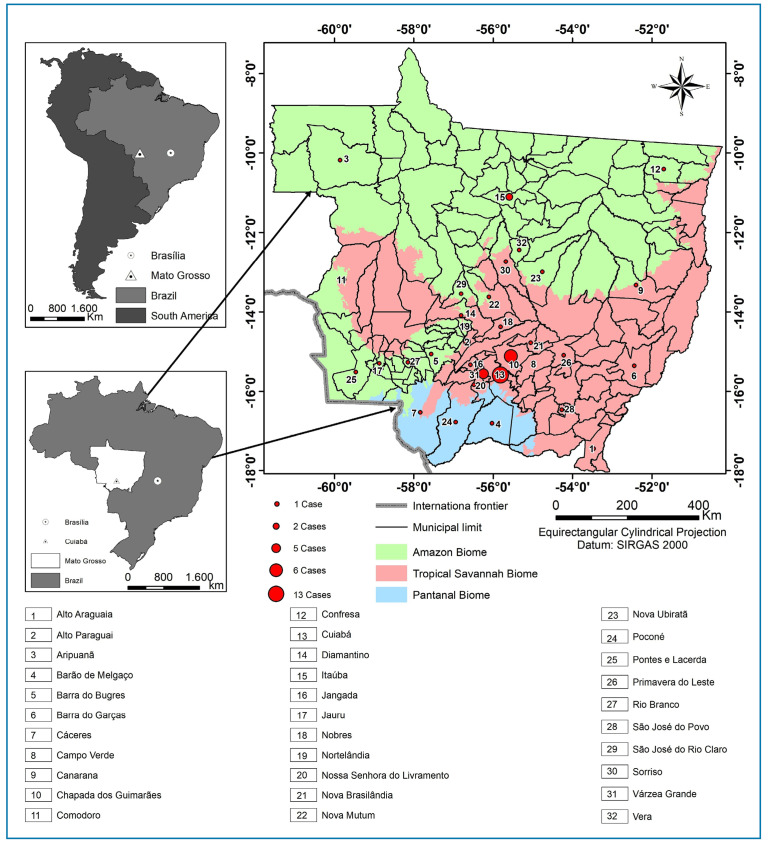
Geographic distribution of the previous location and probable municipality of infection of patients with atypical leishmaniasis treated at HUJM between 2013 and 2023. **Source:** HUJM, 2023.

The source location of atypical cases, that is, the municipality where the infection was reported, is shown on the map of the state of Mato Grosso, divided by municipalities and biomes (Figure 1).

Of the total atypical cases, 43 (91.5%) patients presented with localized CL, two (4.2%) had mucocutaneous leishmaniasis, one case (2.1%) presented with a clinical pattern similar to PKDL, and one distinct case (2.1%) presented with an association between VL and mucocutaneous leishmaniasis lesions.

**FIGURE 2:**
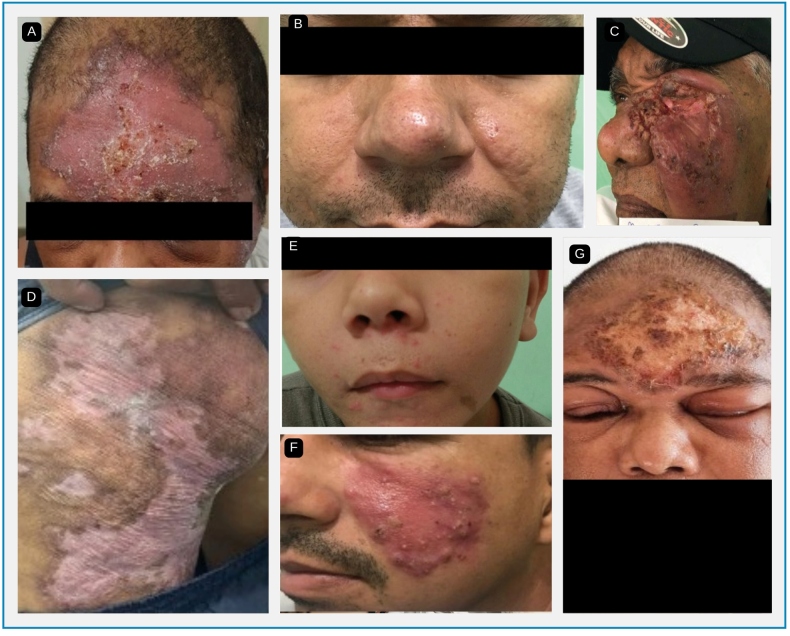
**A:** Erysipeloid form. **B:** Plaque-like form. **C:** Acneiform form. **D:** Psoriasiform form. **E:** Acneiform form. **F:** Plaque-like form. **G:** Erysipeloid form. **Source:** HUJM, 2023.

The cutaneous lesions of CL included erysipeloid, plaque-like, acneiform, and paronychia-like lesions (Figure 2).

**FIGURE 3:**
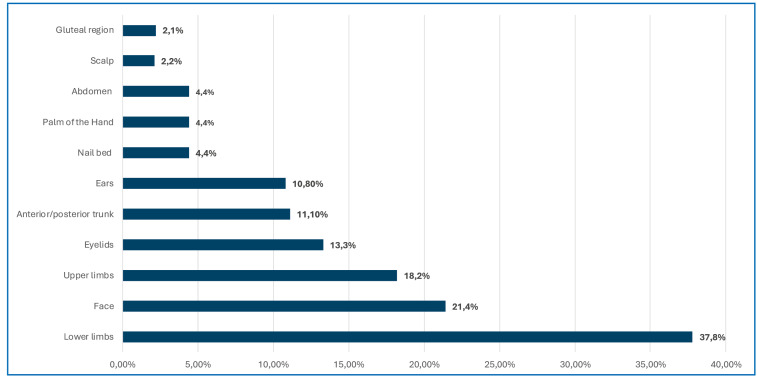
Location of skin lesions in patients with atypical leishmaniasis treated at HUJM between 2013 and 2023. **Source:** HUJM, 2023.

The lower limbs were the most affected areas (37.8%), followed by the face (21.4%), mainly the nasal dorsum and malar, temporal, and frontal regions (Figure 3).

The sociodemographic and epidemiological characteristics of patients with atypical leishmaniasis are presented in Table 1.

**TABLE 1:** Sociodemographic and epidemiological characteristics of patients with atypical leishmaniasis (n= 47).

Characteristics	n (%)
**Sex**	
Male	41 (87,2)
Female	6 (12,8)
**Age range**	
< 20	2 (4,2)
20-29	4 (8,5)
30-39	5 (10,6)
40-49	9 (19,1)
50-59	13 (27,6)
≥ 60	14 (29,8)
Age, median (IQR), years	54 (44-67)
**Occupation**	
Rural worker	12 (25,5)
General services	6 (12,8)
Driver	4 (8,5)
Painter / Bricklayer / Carpenter	4 (8,5)
Retiree	4 (8,5)
Forestry engineer / Geologist	2 (4,3)
Truck driver	2 (4,3)
Mechanic	2 (4,3)
Child	2 (4,3)
Other *	4 (8,5)
Not informed	5 (10,6)

**Legend: IQR**: interquartile range. * Includes educator (n= 1), homemaker (n= 1), barber (n= 1), and administrative manager (n= 1). **Source:** HUJM, 2023.

The median time for lesion evolution was 120 d, and the first quartile was 60 d. Six patients (12.8%) had a history of previous leishmaniasis.

Among the clinical manifestations reported in atypical leishmaniasis cases, lesion pain was the most frequent (34%). The second most frequent manifestation was lymph node enlargement, which accounted for 21.9% of the cases. Other symptoms, such as loss of appetite, weight loss, fever, and diarrhea, were reported less frequently.

Regarding lesion characteristics described in the medical records at the first patient visit, in order of frequency, these included the presence of ulcers in 25 cases (55.3%), elevated edges in 21 (44.7%), infiltration in 12 (25.5%), hematic/yellow crusts in 12 (25.5%), hyperemia, and the presence of fibrin in nine (19.1%). Other less frequently described characteristics were hyaline, purulent, or bloody secretion; granulation tissue; plaque-like lesions; and edema. The terms granulomatous/granular, punctiform lesion, lymph node enlargement, slough, and pustule appeared in one or two cases.

Of the 47 patients identified as atypical, information on direct parasitological tests was available in 40 medical records for scraping (positivity rate, 68%), 42 for aspiration (57%), and 41 for imprint (72.5%). *Leishmania* was detected using PCR in 16 patients, with a detection rate of 100%. Only 10 medical records contained *Leishmania* culture results, with positivity in eight (80%). Two patients were diagnosed by immunohistochemistry and one by lymph node biopsy. In four patients (8.5%), *L. (L.) infantum* was identified and isolated from CL lesions, one of which was PKDL-like, and *L. (V.) braziliensis* was identified in another patient. In the remaining patients, characterization was based solely on the genus.

At the first consultation, 87.2% of patients reported that this was their first episode of leishmaniasis and denied any similar lesion or diagnosis in the past.

Information regarding the history of leishmaniasis treatment prior to arrival at our facility was available in 44 of the 47 medical records. Of these 44 patients, 59% had never been treated before this visit, whereas 15 (34%) had received treatment at another facility.

Among the 15 previously treated patients, 11 (73%) had used Glucantime® (meglumine antimoniate) as their first treatment at another facility, and three (20%) had been prescribed liposomal amphotericin B at the service prior to this consultation. Three patients had completed more than one cycle of Glucantime® at another facility.

Of the 47 patients, information on prior treatment was available for 46, 45 of whom were treated at our facility. In 19 patients, more than one class of medication was prescribed, considering both previous and current treatments. In 20 patients, more than one treatment was prescribed at the facility; among these, 10 had received prior treatment, meaning that these patients received three or more treatments in total.

## DISCUSSION

Leishmaniasis can manifest with a myriad of clinical, immunological, and pathological characteristics influenced by specific factors involving the parasite and host. Its wide geographic distribution, combined with the diversity of vectors present in different climates and biomes, makes leishmaniasis a complex and multifaceted disease that hinders the standardization of methods for effective diagnosis and treatment^11^.

In the present study, among the 1,041 cases of CL registered at HUJM, 47 (4.5%) were classified as atypical, a proportion similar to that observed in other endemic regions. Studies conducted in Bahia reported a frequency of 1.9% atypical cases, whereas in India, approximately 4.2% of CL cases presented with unusual manifestations, especially those associated with *Leishmania donovani* ^3^ ^,^ ^8^ ^,^ ^12^. In Portugal, CL is considered rare and sporadic, and *L. infantum* is the only agent identified in autochthonous cases, a species traditionally associated with VL^13^.

Atypical manifestations of leishmaniasis should not be interpreted merely as rare clinical variations but as part of a dynamic clinical spectrum of the disease. Contemporary studies have indicated changes in parasite tropism and the ability of traditionally visceral species to cause cutaneous forms in immunocompetent individuals. In this context, the identification of *Leishmania (Leishmania) infantum* in cutaneous lesions observed in this study corroborates recent findings described in Brazil and Central America, reinforcing the progressive dissociation between parasite species and classic clinical forms^14^ ^-^ ^16^.

The low proportion of reported atypical cases (4.5%) may be related to underreporting. Furthermore, the absence of standardized diagnostic criteria may lead to misclassification of cases by healthcare staff as typical leishmaniasis.

The analyzed patients presented with varied forms of atypical leishmaniasis, including localized CL (91.5%), which was the predominant form, consistent with national and international reports^2^ ^,^ ^17^. The mucocutaneous form (4.2%) and PKDL-like form (2.1%) were less frequent but clinically relevant, as they complicate diagnosis and therapeutic management. Also noteworthy is the presence of a distinct case involving an association between VL and mucocutaneous leishmaniasis. These presentations were considered atypical in the Brazilian context, where there is no endemic description of PKDL, unlike in regions, such as the Indian subcontinent and East Africa.

It can be hypothesized that the peak number of cases observed in 2018 may be related to climatic factors. During this period, the state of Mato Grosso experienced temperatures above the historical average and reduced rainfall, which may have favored the proliferation of phlebotomine sand flies and influenced the transmission of leishmaniasis, although a direct causal relation could not be established in this study^6^ ^,^ ^18^.

The demographic profile of the cases registered at HUJM was similar to that reported by PAHO^19^. A higher proportion of male patients in the 20-59-year age range was observed, which is consistent with the findings of other studies. As expected, the main occupational profile of patients with atypical leishmaniasis was that of rural workers^11^ ^,^ ^19^ ^,^ ^20^.

The concentration of cases in patients aged 50-59 years suggests that immunosenescence may influence the host response to the parasite, favoring atypical manifestations. Furthermore, this response may be associated with factors, such as occupational exposure and outdoor recreational activities, which increase contact with the vector and lead to greater demand for medical care for unusual symptoms. Moreover, the presence of comorbidities may increase vulnerability to infection. These factors make this population more susceptible to both infection and atypical manifestations of the disease^19^ ^,^ ^20^.

The most frequent clinical presentations included erysipeloid and acneiform lesions, predominantly on the lower limbs (37.8%) and face (21.4%). This differs from the findings of other studies, in which atypical lesions were more frequent in the upper limbs (18.1%). This indicates that exposed areas, such as the face and legs, are the most vulnerable to skin lesions, possibly owing to greater exposure to the vector^21^ ^-^ ^23^. Eyelid involvement, although infrequent, was higher than that described in other series, probably owing to the mobility and sensitivity of the eyelids, which make it difficult for the vector to bite^9^.

This study showed that conventional parasitological examinations, such as scraping, aspiration, and imprinting, were widely used; however, PCR was applied in a limited number of cases, limiting the precise identification of the species involved. Nevertheless, the detection of *L. (L.) infantum* in immunocompetent patients reinforces the need for the systematic incorporation of molecular methods in the diagnosis of leishmaniasis, as evidenced by studies conducted in other countries^14^ ^,^ ^16^ ^,^ ^24^.

In India, the presence of *L. donovani* has been observed in cases of CL^24^, as well as in Honduras, where *L. infantum chagasi* has been identified as the agent of atypical forms of CL^16^. *L. infantum* can cause CL, although more frequently in patients with immunosuppression^14^.

Most cases were concentrated in the tropical savannah biome (Cerrado), particularly in the central and southeastern regions of Mato Grosso. The Amazon biome in the north and Pantanal biome in the southwest had fewer municipalities with recorded cases of atypical leishmaniasis.

The central region of the state, where Cuiabá is located, had the highest number of cases. This may be associated with factors, such as population density and greater exposure to transitional areas between biomes that may harbor the leishmaniasis vector. This distribution also suggests that areas with agricultural activity and deforestation, which are common in the Cerrado, may be linked to increased exposure to the disease^25^.

The pattern of clinical manifestations of atypical leishmaniasis suggests that it may present with various localized and/or systemic symptoms. Symptoms reported by patients, such as lesion pain, lymphadenopathy, and fever, were the most prevalent, suggesting an inflammatory nature of the disease^8^ ^,^ ^15^.

The presence of reported systemic symptoms, such as weight loss and hepatosplenomegaly, observed in a smaller proportion of patients with CL, indicates that some cases of atypical leishmaniasis may present with systemic manifestations that mimic the visceral form, making differential diagnosis difficult, especially in endemic areas.

The therapeutic failure rate was high, with 54% of patients requiring retreatment; of these, 95% used more than one class of medication. Similar results have been reported in the literature; for example, in Bahia, high failure rates with Glucantime® have been described, requiring a switch to amphotericin B^8^. In Honduras, atypical cases have shown partial resistance to antimony, requiring prolonged regimens^26^.

Atypical CL (ACL) is an underestimated clinical entity that is difficult to diagnose, and its occurrence has been increasing in endemic regions such as the Brazilian Midwest^19^. The findings of this study highlight the clinical diversity and diagnostic ambiguity of ACL, with presentations ranging from erysipeloid to PKDL-like forms, as well as its possible association with environmental and climatic factors that may influence vector transmission dynamics, although direct causal relations could not be established in this study.

The treatment failure rate, especially with the initial use of antimony and need for escalation, reinforces the necessity of creating or revising treatment protocols. The detection of *Leishmania infantum* in skin lesions, even in immunocompetent individuals, is a pattern rarely found in the national and international literature. This highlights the changing pathogenic profile of this disease and reinforces the need for early species identification using molecular methods.

Given the scenario of climate change, increased human occupation in transmission areas, and the absence of a diagnostic consensus, ACL should not simply be considered a rare clinical variant but rather a condition of growing relevance in endemic areas, with implications for epidemiological surveillance and clinical management.

These results reinforce the importance of establishing standardized diagnostic criteria, mapping the involved species through molecular tests, and developing specific therapeutic guidelines, aiming to enable early diagnosis and management of the disease to ensure better clinical outcomes for patients.

## Data Availability

Research data is only available upon request.
